# Celiac disease increases the risk of pulmonary arterial hypertension: A multivariable Mendelian randomization and mediation analysis

**DOI:** 10.1097/MD.0000000000048334

**Published:** 2026-04-10

**Authors:** Meng Wu, Weidong Song, Xin Guo, Zhongyuan Lu, Jin Yang, Zhimei Hu

**Affiliations:** a Outpatient Department, The 903rd Hospital of the Joint Logistic Support Force, Hangzhou, Zhejiang, China; b Thoracic Surgery Department, Ganzhou Cancer Hospital, Ganzhou, Jiangxi,China; c Jiangxi Provincial People’s Hospital, The First Affiliated Hospital of Nanchang Medical College, Nanchang, Jiangxi, China.

**Keywords:** autoimmunity, celiac disease, Mendelian randomization, pulmonary arterial hypertension, systemic lupus erythematosus

## Abstract

Celiac disease (CeD) has been implicated in several autoimmune and vascular disorders, but its causal role in pulmonary arterial hypertension (PAH) remains uncertain. Summary-level genome-wide association study data for all traits were obtained from the FinnGen consortium and the genome-wide association study catalog, 2 publicly accessible databases integrating genetic data with health registry information. A 2-sample Mendelian randomization (MR) approach was employed to evaluate the causal effect of CeD on PAH. Univariable MR was conducted initially, and the results were validated through meta-analysis based on 2 independent cohorts. To control for possible confounding influences, multivariable MR was applied with adjustments for obesity and smoking. Mediation analyses were performed to determine whether autoimmune disorders mediated the observed association. Heterogeneity and pleiotropy were assessed to ensure the robustness of the findings. A significant causal association between CeD and PAH was identified in the univariable MR analysis (discovery cohort: odds ratio [OR] of inverse-variance weighted [IVW] [OR_IVW_], 1.148; 95% confidence interval [CI], 1.027–1.282; *P* = .015; replication cohort: OR_IVW_, 1.112; 95% CI, 1.043–1.186; *P* = .001), and this relationship was further supported by the meta-analysis of both cohorts (OR_IVW_, 1.136; 95% CI, 1.065–1.212; *P* < .001). The association remained statistically significant after adjustment for obesity and smoking in multivariable MR (OR_IVW_, 1.138; 95% CI, 1.011–1.281; *P* < .032), suggesting that the effect of CeD on PAH is independent of these factors. Among the AIDs examined, only systemic lupus erythematosus exhibited a meaningful mediating role in this causal pathway (mediation proportion [95% CI]: 30.4% [3.6%–57.2%], *P* < .023). No evidence of heterogeneity or horizontal pleiotropy was detected across all MR analyses (all *P* > .050). Genetic evidence supports that CeD increases the risk of PAH, with systemic lupus erythematosus potentially acting as a mediator. These findings provide new insights into shared autoimmune mechanisms underlying PAH and suggest that immune regulation may represent a potential therapeutic target.

## 1. Introduction

Celiac disease (CeD) is a chronic, immune-mediated enteropathy induced by gluten ingestion in genetically predisposed individuals.^[1,2]^ It is primarily characterized by villous atrophy of the small intestinal mucosa,^[3,4]^ resulting in impaired nutrient absorption and a broad spectrum of extraintestinal manifestations,^[5–7]^ including metabolic, neurological, and musculoskeletal abnormalities. Recent research has increasingly highlighted that CeD is not limited to localized intestinal pathology, but constitutes a systemic autoimmune disorder with widespread immunological consequences.^[8,9]^ The persistent activation of both innate and adaptive immune responses, accompanied by chronic inflammation, could lead to alterations in multiple organ systems beyond the gut. Notably, CeD has been associated with endothelial dysfunction, vascular inflammation, and dysregulation of immune signaling pathways, indicating potential involvement in cardiovascular and pulmonary pathologies.^[10]^ The frequent co-occurrence of CeD with other immune-mediated conditions further suggests that common immunogenetic pathways, such as HLA-related antigen presentation and cytokine network dysregulation,^[11,12]^ may underlie its multisystem involvement. Understanding these systemic effects is crucial for elucidating the broader clinical impact of CeD and for identifying potential mechanisms linking it to secondary complications, including vascular and autoimmune disorders.

Pulmonary arterial hypertension (PAH) is a progressive and life-threatening disorder, defined by sustained elevation of pulmonary arterial pressure and structural remodeling of the pulmonary vasculature.^[13]^ Despite therapeutic advances, PAH remains associated with high morbidity and mortality.^[14,15]^ Accumulating data indicate that immune dysregulation and chronic inflammation are central to PAH pathogenesis, facilitating endothelial dysfunction, vascular smooth muscle proliferation, and remodeling of pulmonary vessels. The high prevalence of PAH in systemic autoimmune diseases (AIDs), including systemic sclerosis (SSc), systemic lupus erythematosus (SLE), and Sjögren syndrome (SS), further underscores the immunological basis of the disorder.^[16–18]^ These observations raise the possibility that CeD, as a systemic autoimmune condition, may predispose individuals to PAH via shared immunopathogenic or inflammatory pathways.

Although case reports and observational studies have documented the co-occurrence of CeD and PAH,^[19]^ the causal relationship remains unclear. Traditional observational designs are susceptible to confounding, selection bias, and reverse causation, limiting their ability to establish causality. Mendelian randomization (MR) analysis,^[20–22]^ which utilizes genetic variants as instrumental variables (IVs), provides a robust approach to infer causality while minimizing these sources of bias.

In this study, a 2-sample MR framework was employed to evaluate the causal effect of CeD on PAH. This comprehensive analytical approach aims to provide rigorous genetic evidence supporting a causal role of CeD in PAH and to elucidate underlying autoimmune mechanisms, thereby contributing to a deeper understanding of immune-mediated vascular pathology and informing potential strategies for risk assessment and intervention.

## 2. MR design

**Figure 1.**
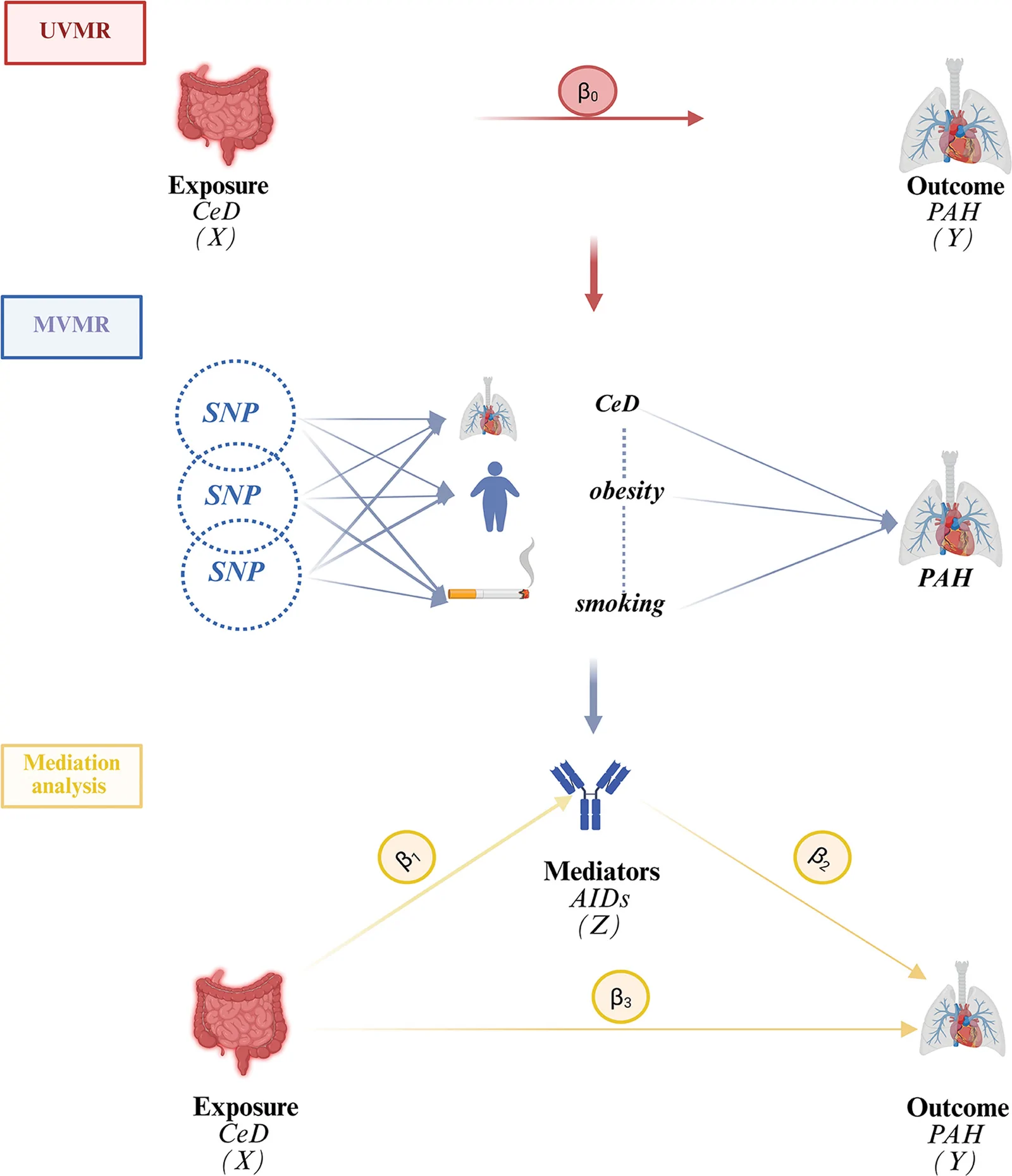
Overview diagram of this MR study evaluating the causal effect of CeD on PAH. AID = autoimmune diseases, CeD = celiac disease, PAH = pulmonary arterial hypertension, MVMR = multivariable Mendelian randomization, SNP = single nucleotide polymorphism, UVMR = univariable Mendelian randomization.

A univariable MR (UVMR) design based on the core assumptions of MR was applied to investigate the causal relationship between CeD and PAH. To enhance robustness, a meta-analysis of 2 independent cohorts was performed to validate the causal estimates. Multivariable MR (MVMR) analysis^[23]^ was further conducted to adjust for potential confounders including obesity and smoking, which are known to influence vascular health. Additionally, mediation analyses were employed to explore whether AIDs mediate the effect of CeD on PAH (Fig. 1).

### 2.1. Data collection and description

Summary-level genome-wide association study (GWAS) data for all traits were obtained from 2 publicly accessible databases. Data for CeD in the discovery cohort (finngen_R11_K11_COELIAC) and SSc (finngen_R11_M13_SYSTSLCE) were obtained from the FinnGen consortium (https://www.finngen.fi/en),^[24]^ which integrates genetic data from Finnish biobanks with nationwide health registry information. GWAS data for CeD in the replication cohort (GCST000612), PAH (GCST0072288), obesity (GCST001475), smoking (ukb-a-17), SLE (GCST90018917), and SS (GCST90013879) were derived from the GWAS Catalog, a publicly curated and standardized repository of published GWAS studies (https://www.ebi.ac.uk/gwas/).^[25]^ All datasets were derived from individuals of European ancestry, and detailed information is summarized in Table 1.

**Table 1:** Overview of the GWAS summary data utilized in this MR study.

Exposure/outcome/mediation	ID of GWAS	Sample size	Year	Population
CeD (discovery cohort)	GCST000612	15,283	2010	European
CeD (replication cohort)	finngen_R11_K11_COELIAC	438,467	2024	European
PAH	GCST0072288	11,744	2018	European
Obesity	GCST001475	13,848	2012	European
Smoking	ukb-a-17	310,749	2017	European
SSc	finngen_R11_M13_SYSTSLCE	440,192	2024	European
SLE	GCST90018917	482,911	2021	European
SS	GCST90013879	407,746	2021	European

CeD = celiac disease, GWAS = genome-wide association study, PAH = pulmonary arterial hypertension, SLE = systemic lupus erythematosus, SS = Sjogren syndrome, SSc = systemic sclerosis.

### 2.2. Selection of genetic proxies

**Figure 2.**
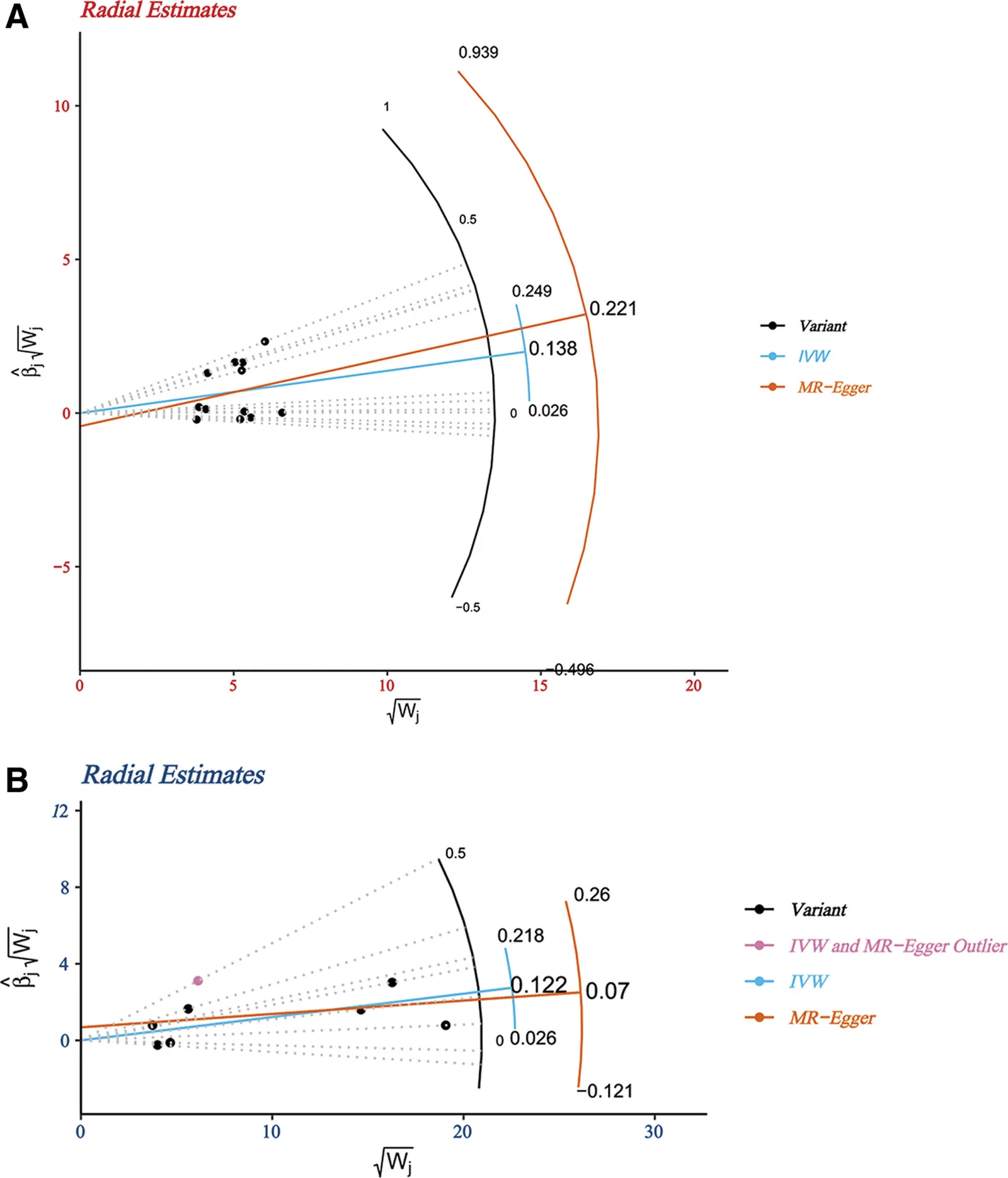
Radial plots illustrating individual outlier SNPs identified by radial MR in this MR study evaluating the causal effect of CeD on PAH. (A) Radial plot illustrating individual outlier SNPs identified by radial MR in discovery cohort. (B) Radial plot illustrating individual outlier SNPs identified by radial MR in replication cohort. CeD = celiac disease; IVW = inverse-variance weighted, MR = Mendelian randomization, PAH = pulmonary arterial hypertension, SNPs = single nucleotide polymorphism.

Single nucleotide polymorphisms (SNPs) strongly associated with CeD were initially selected using a genome-wide significance threshold of *P* < 5 × 10^−8^. To minimize the influence of linkage disequilibrium, SNPs were clumped with an *r*^2^ threshold of 0.001 and a window of 10,000 kb. SNPs with an *F* statistic ≤10 were excluded to remove weak instruments (*F* = (β/SE)^2^).^[26]^ Outlier SNPs were subsequently identified using Radial MR (Fig. 2, Table 2), and SNPs showing reverse-direction effects were filtered out based on the MR Steiger test. The remaining SNPs were retained as genetic IVs for CeD and utilized in subsequent MR analyses. Detailed information on the selected instruments is provided in Table S1, Supplemental Digital Content, https://links.lww.com/MD/R669.

**Table 2:** Outlier SNPs identified by radial MR.

Exposure	Outcome	Outlier SNPs	
		IVW	MR-Egger
CeD (discovery cohort)	PAH	NA	NA
CeD (replication cohort)	PAH	rs13093110	rs13093110

CeD = celiac disease, IVW = inverse-variance weighted, MR = Mendelian randomization, NA = not available, PAH = pulmonary arterial hypertension, SNPs = single nucleotide polymorphisms.

### 2.3. Statistical methods

In the UVMR analysis, the causal relationship between CeD and PAH was primarily assessed using the inverse-variance weighted (IVW) method,^[27]^ complemented by MR-Egger^[28]^ and weighted median approaches.^[29]^ Considering the continuous development of MR methodologies, additional analyses were conducted using the contamination mixture,^[30]^ MR robust adjusted profile score,^[31]^ and debiased inverse-variance weighted methods to enhance robustness.^[32]^ For the MVMR analysis, IVW, MR-Egger, least absolute shrinkage and selection operator,^[33]^ and weighted median methods were employed to evaluate the independent causal effect after adjusting for potential confounders.

**Figure 3.**
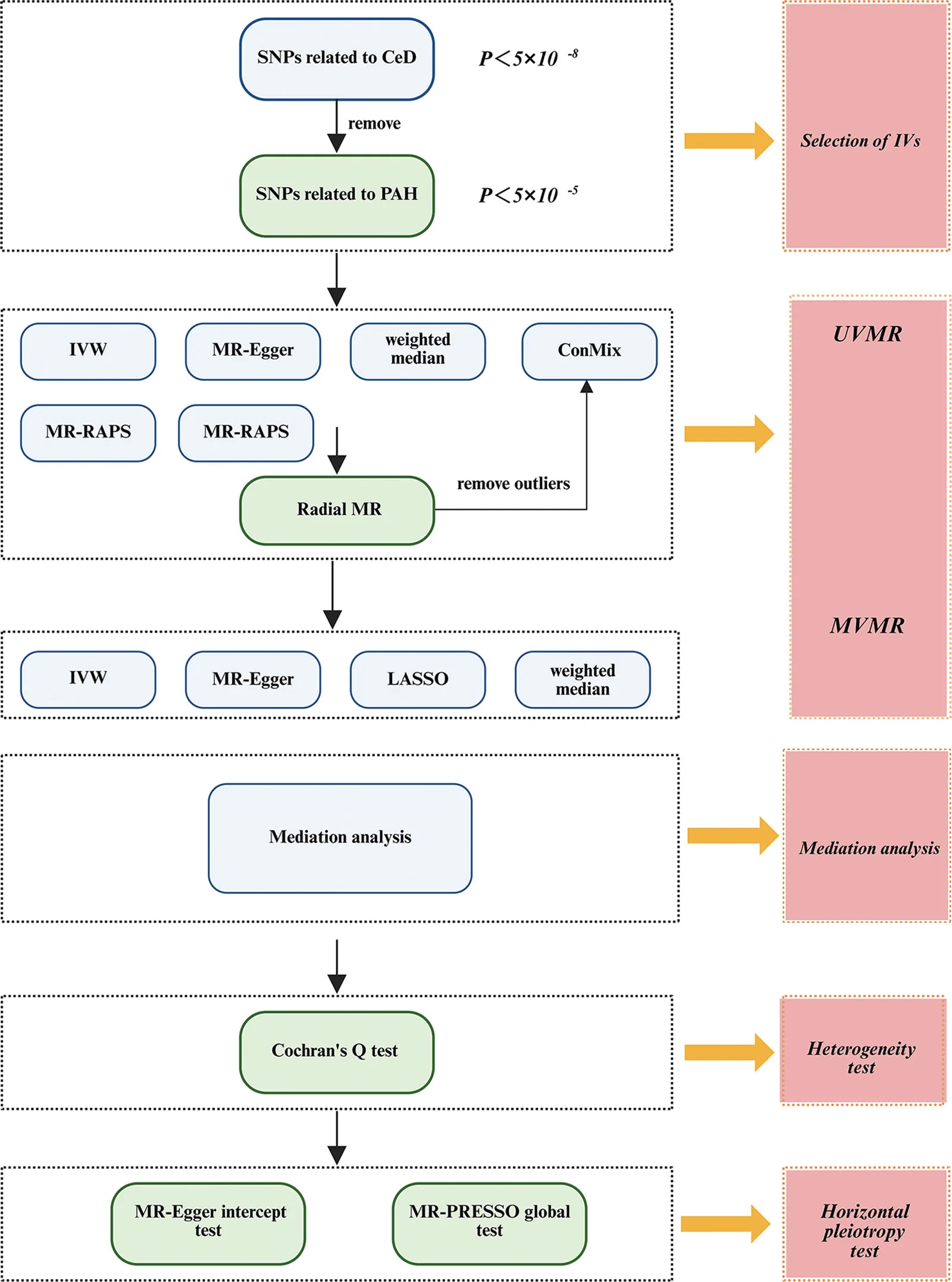
Flow diagram of this MR study evaluating the causal effect of CeD on PAH. CeD = celiac disease, ConMix =, contamination mixture, dIVW = debiased inverse-variance weighted method, IVs = instrumental variables, IVW = inverse-variance weighted, LASSO = least absolute shrinkage and selection operator, MR = Mendelian randomization, MR-PRESSO = Mendelian randomization pleiotropy residual sum and outlier, MR-RAPS = Mendelian randomization robust adjusted profile score, MVMR = multivariable Mendelian randomization, PAH = pulmonary arterial hypertension, SNPs = single nucleotide polymorphisms, UVMR = univariable Mendelian randomization.

To assess the mediating role of AIDs between CeD and PAH, we employed a 2-step mediation analysis. In the 1st step, we evaluated the causal effect of the exposure variable (CeD) on the mediator variable (AIDs) by calculating $\beta_{1}$, which measures the effect of CeD on AIDs. If $\beta_{1}$ is significant, it indicates that CeD has a significant effect on AIDs. In the 2nd step, we evaluated the causal effect of the mediator variable (AIDs) on the outcome variable (PAH), by calculating $\beta_{2}$, which measures the effect of AIDs on PAH, while controlling for the effect of CeD. If $\beta_{2}$ is significant, it suggests that AIDs mediate the relationship between CeD and PAH. The total effect ($\beta_{3}$) reflects the overall impact of the exposure variable (CeD) on the outcome variable (PAH). The mediation effect is estimated by calculating the indirect effect ($\beta_{1}\times \beta_{2}$), which represents the indirect effect of CeD on PAH through AIDs. If the indirect effect is significant, it indicates the presence of a mediation effect. The direct effect ($\beta_{direct}$) can be calculated as $\beta_{3}-\beta_{indirect}$, representing the direct effect of the exposure on the outcome. Additionally, the proportion mediated is calculated as the ratio of the indirect effect to the total effect ($proportion\;mediated=\frac{\beta_{indirect}}{\beta_{3}}$), reflecting the proportion of the total effect that is mediated. This method is simple and intuitive but assumes that each regression model is independent, which may be influenced by omitted variables or insufficient control, making it suitable for simpler mediation models. To assess the robustness and reliability of the findings, several sensitivity analyses were conducted to address potential sources of bias and to ensure the validity of the results. First, heterogeneity among the in IVs was tested using Cochran *Q* test,^[34]^ which helps determine whether there are significant differences in the associations of the IVs with the outcome. To evaluate the presence of horizontal pleiotropy, 2 different approaches were employed, including the MR pleiotropy residual sum and outlier global test and the MR-Egger intercept test. The MR pleiotropy residual sum and outlier global test examines the overall impact of pleiotropy on the MR analysis, while the MR-Egger intercept test evaluates whether the intercept differs significantly from zero, which could indicate the directional pleiotropy (Fig. 3). Additionally, funnel plots were generated to visually assess the symmetry of SNP distributions. Any asymmetry observed in the funnel plot could suggest the presence of heterogeneity. *P* < .05 was considered statistically significant.

## 3. Results

### 3.1. Causal association between CeD and PAH in UVMR

**Figure 4.**
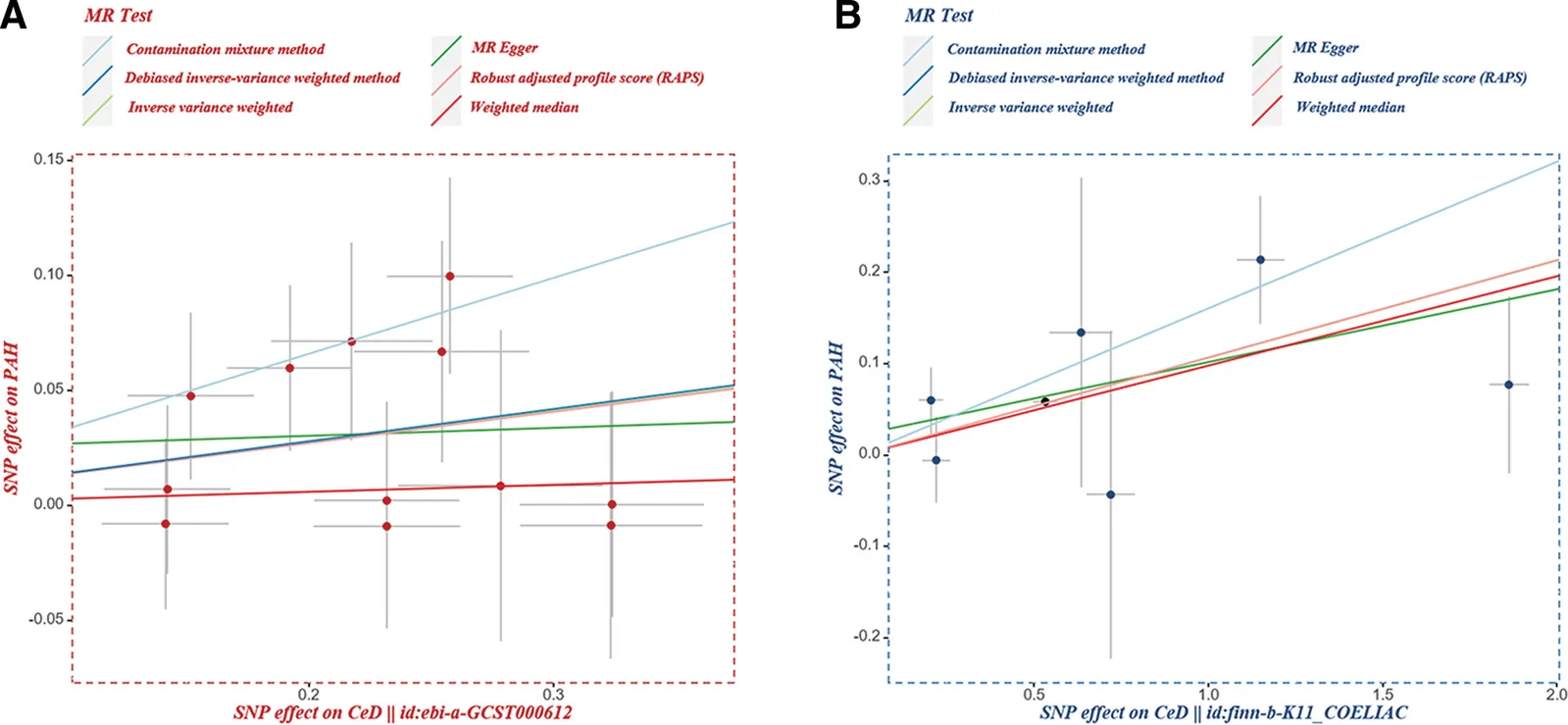
Scatter plots illustrating the causal effect of CeD on PAH in UVMR. (A) Scatter plot illustrating the causal effect of CeD of discovery cohort on PAH in UVMR. (B) Scatter plot illustrating the causal effect of CeD of replication cohort on PAH in UVMR. CeD = celiac disease, MR, Mendelian randomization, PAH = pulmonary arterial hypertension, UVMR = univariable Mendelian randomization.

**Figure 5.**
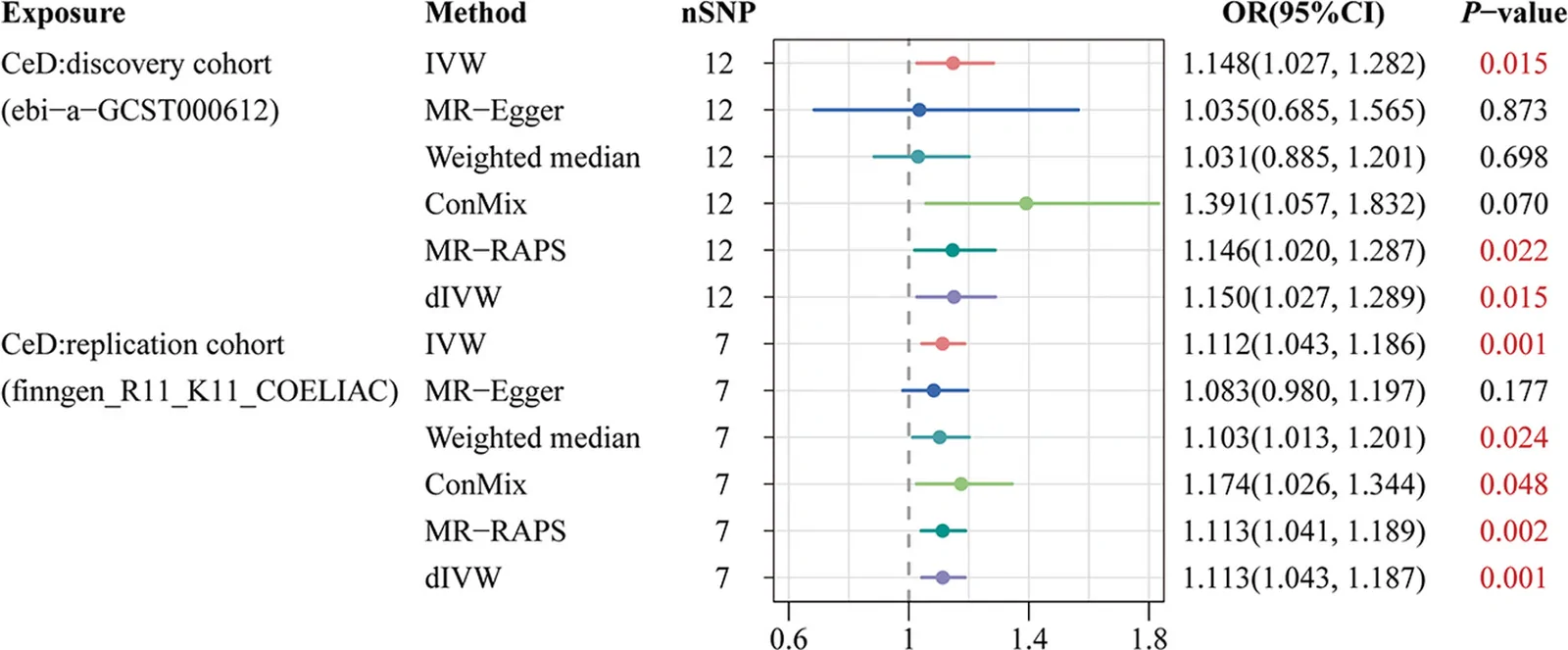
Forest plot illustrating the causal effect of CeD on PAH in UVMR. 95% CI = 95% confidence interval, CeD = celiac disease, ConMix =, contamination mixture, dIVW = debiased inverse-variance weighted method, IVW = inverse-variance weighted, MR = Mendelian randomization, MR-RAPS = Mendelian randomization robust adjusted profile score, OR = odds ratio, PAH = pulmonary arterial hypertension, SNP = single nucleotide polymorphism, UVMR = univariable Mendelian randomization..

**Figure 6.**
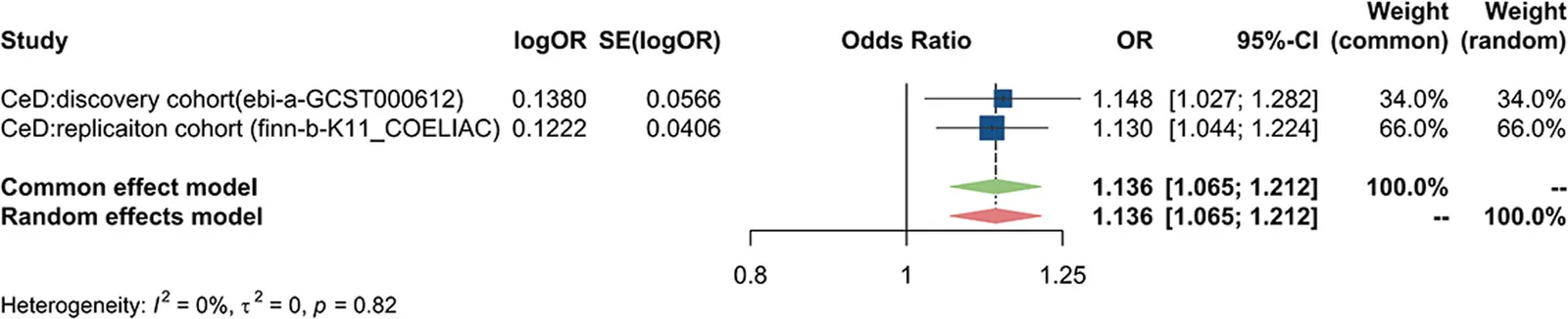
Forest plot illustrating the combined causal effects of CeD on PAH from the discovery and replication cohorts in the UVMR analysis. 95% CI = 95% confidence interval, CeD = celiac disease, OR = odds ratio, PAH = pulmonary arterial hypertension, UVMR = univariable Mendelian randomization.

A significant causal association between CeD and PAH was identified in UVMR analysis (discovery cohort: odds ratio [OR] of IVW [OR_IVW_], 1.148; 95% confidence interval [CI], 1.027–1.282; *P* = .015; replication cohort: OR_IVW_, 1.112; 95% CI, 1.043–1.186; *P* = .001) (Figs. 4 and 5). Furthermore, meta-analysis combining results from both the discovery and replication cohorts yielded consistent and statistically significant estimates (OR_IVW_, 1.136; 95% CI, 1.065–1.212; *P*<.001) (Fig. 6), reinforcing the reliability of the observed causal effect.

### 3.2. Causal association between CeD and PAH in MVMR

**Figure 7.**
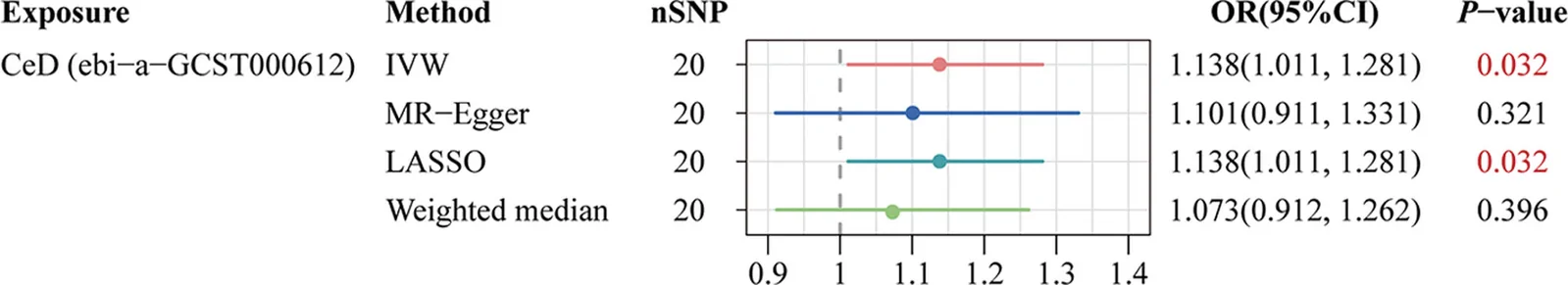
Forest plot illustrating the causal effect of CeD on PAH in MVMR adjusting for the confounding effect. 95% CI = 95% confidence interval; CeD = celiac disease, IVW = inverse-variance weighted, LASSO = least absolute shrinkage and selection operator, MVMR = multivariable Mendelian randomization, OR = odds ratio, PAH = pulmonary arterial hypertension, SNP = single nucleotide polymorphism.

After adjustment for obesity and smoking in the MVMR analysis, the association between CeD and PAH persisted with statistical significance (OR_IVW_, 1.138; 95% CI, 1.011–1.281; *P* < .032) (Fig. 7), suggesting that the effect of CeD on PAH is not attributable to these metabolic or lifestyle-related factors.

### 3.3. Mediating effect of AIDs in the causal association *t* of CeD on PAH

**Figure 8.**
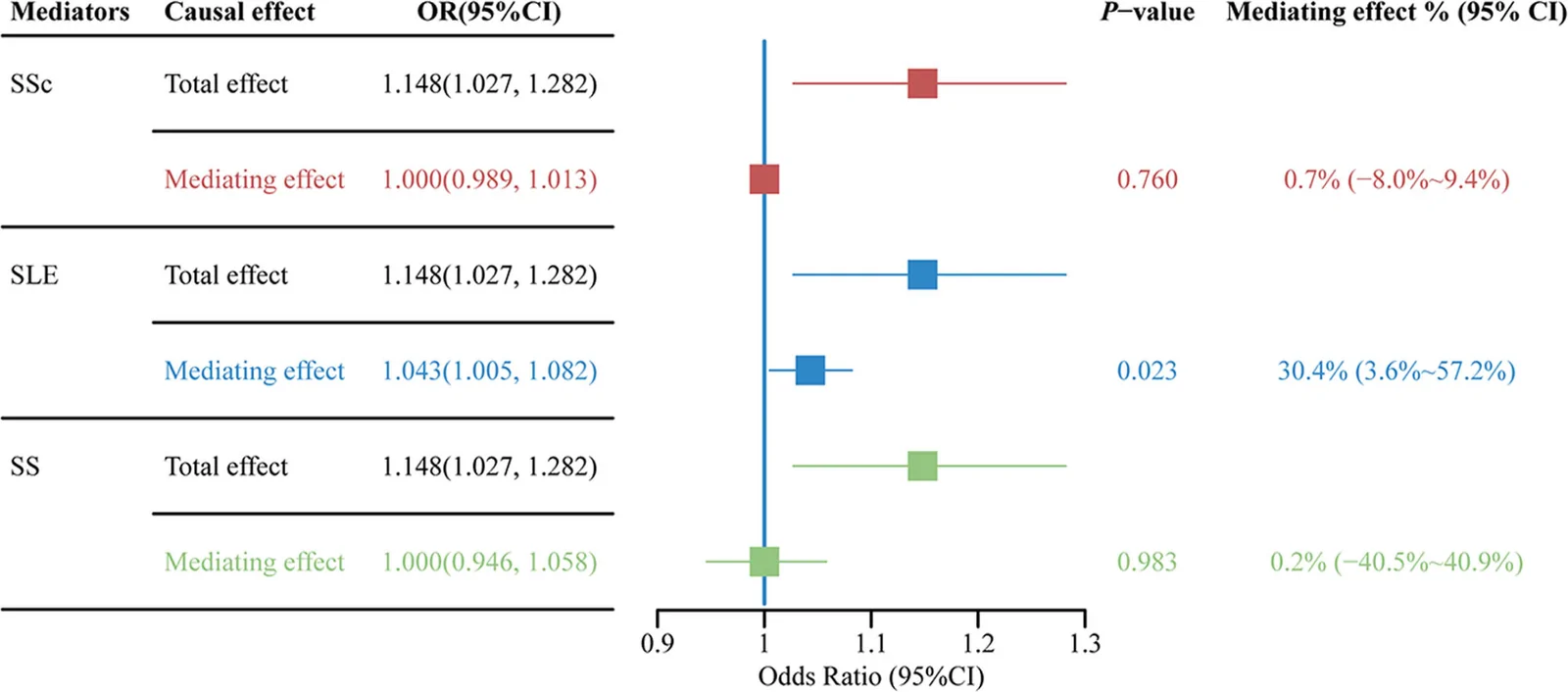
Forest plot illustrating the mediating effect of AIDs on the causal link between CeD and PAH. 95% CI = 95% confidence interval, AIDs = autoimmune diseases, CeD = celiac disease, OR = odds ratio, PAH = pulmonary arterial hypertension, SLE = systemic lupus erythematosus, SS = Sjogren syndrome, SSc = systemic sclerosis.

Among the AIDs assessed, only SLE demonstrated a significant mediating effect in the causal pathway between CeD and PAH (mediation proportion [95% CI]: 30.4% [3.6%–57.2%], *P* < .023), whereas SSc and SS showed no significant mediation effects (SSc: mediation proportion [95% CI]: 0.7% [−8.0% to 9.4%], *P* = .760; SS: mediation proportion [95% CI]: 0.2% [−40.5% to 40.9%], *P* = .983) (Fig. 8).

### 3.4. The result of sensitivity analysis

No evidence of heterogeneity was observed across all MR analyses (all *P* > .050). Similarly, no indication of horizontal pleiotropy was detected (all *P* > .050) (Table 3).

**Table 3:** The results of the sensitivity analysis in this MR analysis.

Exposure	Cochran *Q* test		Egger intercept test		MR-PRESSO
	(*P* value) IVW	(*P* value) MR-Egger	Egger intercept	*P* value	*P* value of global test
CeD (discovery cohort)	.635	.570	0.024	.623	.636
CeD (replication cohort)	.499	.430	0.022	.523	.461

CeD = celiac disease, IVW = inverse-variance weighted, MR = Mendelian randomization, MR-PRESSO = Mendelian randomization pleiotropy residual sum and outlier.

## 4. Discussion

In the present study, we provide comprehensive genetic evidence supporting a causal association between CeD and PAH through MR analyses. UVMR indicated that CeD may act as a potential risk factor for PAH, and these findings were reinforced by meta-analysis of 2 independent cohorts. The association remained statistically significant in MVMR analyses after adjusting for established confounders such as obesity and smoking, suggesting that the effect of CeD on PAH is independent of these common vascular risk factors. Moreover, mediation analyses revealed that SLE, but not SSc or SS, may partially mediate this causal pathway. Sensitivity analyses demonstrated no evidence of heterogeneity or horizontal pleiotropy, supporting the robustness and reliability of our findings.

Existing literature on the association between CeD and PAH is extremely limited, with most reports being individual case studies.^[19]^ No large-scale observational or mechanistic studies have systematically evaluated this relationship. Consequently, the direction and magnitude of the association have remained uncertain. By using genetic instruments in an MR framework, the present study overcomes these limitations and provides the 1st robust evidence suggesting a causal link between CeD and PAH. The persistence of the association after adjustment for obesity and smoking further indicates that the effect of CeD on PAH is unlikely to be explained solely by traditional cardiometabolic risk factors, but may instead reflect underlying autoimmune or inflammatory processes.

Several potential mechanisms may underlie the observed causal relationship between CeD and PAH. Chronic intestinal inflammation in CeD can lead to systemic immune activation, resulting in elevated circulating proinflammatory cytokines such as interleukins, tumor necrosis factor-α, and interferons,^[35–37]^ which are known to induce endothelial dysfunction, promote vascular smooth muscle proliferation, and contribute to pulmonary vascular remodeling.^[38,39]^ In addition, CeD-associated nutrient malabsorption and deficiencies,^[40]^ including iron, folate, and fat-soluble vitamins, may exacerbate oxidative stress, impair nitric oxide bioavailability, and compromise endothelial repair processes, further predisposing individuals to vascular injury. Genetic predisposition shared between CeD and other autoimmune-mediated vascular disorders^[41]^ may also amplify susceptibility to PAH, as overlapping immunogenetic pathways could facilitate aberrant immune responses targeting the pulmonary vasculature. Moreover, CeD may increase the risk of developing secondary systemic autoimmune diseases,^[42]^ as evidenced by the partial mediation effect of SLE identified in our study, suggesting that persistent immune dysregulation may amplify vascular inflammation and remodeling through mechanisms such as immune complex deposition, complement activation, and autoantibody-mediated endothelial damage. Chronic systemic inflammation associated with CeD may further activate fibroblast proliferation and extracellular matrix deposition within pulmonary arterioles, leading to increased pulmonary vascular resistance, while endothelial dysfunction may be compounded by dysregulated angiogenic signaling and impaired endothelial progenitor cell function, which have been implicated in PAH pathogenesis. Beyond immune-mediated mechanisms, alterations in gut microbiota and intestinal permeability observed in CeD^[43]^ may contribute to systemic inflammation and vascular dysfunction, as translocation of microbial products into the circulation can stimulate innate immune responses and promote vascular endothelial activation. Collectively, these interrelated pathways – including chronic systemic inflammation, immune dysregulation, nutrient deficiency, vascular remodeling, and gut-derived immune activation – provide a plausible biological basis for the causal link between CeD and PAH, highlighting the complex interplay between intestinal autoimmunity and pulmonary vascular pathology.

It is noteworthy that several limitations of the present study should be acknowledged.^[44,45]^ First, the analyses were conducted mainly in populations of European ancestry, which may limit generalizability to other populations, and further studies are needed to evaluate ethnic-specific effects. Mediation analysis provides a framework to explore intermediate mechanisms but cannot fully resolve complex temporal and biological interactions among CeD, systemic AIDs, and PAH. Although SLE was identified as a potential mediator, the precise molecular and cellular mechanisms remain unclear, warranting functional and longitudinal studies to confirm its role and explore other immune pathways. While MR analysis is a robust tool for causal inference, it assumes that genetic instruments affect the outcome solely through the exposure, and violations of this assumption could introduce bias. Additionally, the clinical relevance of the observed effect sizes requires cautious interpretation and further validation. Finally, prospective cohorts integrating multi-omics data, detailed phenotyping, and longitudinal follow-up are needed to translate these genetic findings into risk stratification, early detection, and preventive strategies, which will be critical for confirming the causal role of CeD in PAH and identifying potential intervention targets. Such studies may also provide valuable insights into the temporal progression of disease, clarify underlying mechanisms, and guide the development of targeted therapies for high-risk individuals.

In summary, this study provides robust evidence that CeD is a causal risk factor for PAH and suggests that SLE may partially mediate this association. These findings advance our understanding of autoimmune and inflammatory mechanisms linking gastrointestinal and pulmonary vascular disorders. Clinically, they highlight the need for careful cardiovascular and autoimmune monitoring in patients with CeD and underscore the potential for early intervention to mitigate PAH risk in susceptible individuals. Future studies integrating multi-omics, functional experiments, and longitudinal clinical data are warranted to further elucidate the underlying pathways and identify potential targets for prevention and therapy.

## 5. Conclusion

Our study provides genetic evidence supporting a causal relationship between CeD and PAH, with SLE partially mediating this effect. These findings highlight the potential role of immune-mediated mechanisms in PAH development among individuals with CeD and underscore the importance of autoimmune surveillance and early intervention to mitigate pulmonary vascular risk.

## Author contributions

**Conceptualization:** Zhimei Hu.

**Investigation:** Zhongyuan Lu.

**Methodology:** Zhongyuan Lu.

**Visualization:** Jin Yang.

**Writing – original draft:** Meng Wu, Weidong Song, Xin Guo.

## Supplementary Material


